# Latent Tuberculosis Infection and Associated Factors among Health Care Workers in Kigali, Rwanda

**DOI:** 10.1371/journal.pone.0124485

**Published:** 2015-04-28

**Authors:** Claude Rutanga, David W. Lowrance, John E. Oeltmann, Grace Mutembayire, Matt Willis, Claude Bernard Uwizeye, Ruton Hinda, Chitou Bassirou, Steve Gutreuter, Michel Gasana

**Affiliations:** 1 Division of Tuberculosis and Other Respiratory Communicable Diseases Rwanda Biomedical Centre, Kigali, Rwanda; 2 Division of Global HIV/AIDS, Centers for Disease Control and Prevention, Kigali, Rwanda; 3 Division of Tuberculosis Elimination, Centers for Disease Control and Prevention, Atlanta, United States of America; 4 Division of HIV-AIDS, Sexually Transmitted Infections, and Other Blood Borne Diseases, Rwanda Biomedical Centre, Kigali, Rwanda; 5 Division of Global HIV/AIDS, Centers for Disease Control and Prevention, Atlanta, United States of America

## Abstract

**Introduction:**

Data are limited regarding tuberculosis (TB) and latent TB infection prevalence in Rwandan health facilities.

**Methods:**

We conducted a cross-sectional survey among healthcare workers (HCWs) in Kigali during 2010. We purposively selected the public referral hospital, both district hospitals, and randomly selected 7 of 17 health centers. School workers (SWs) from the nearest willing public schools served as a local reference group. We tested for latent TB infection (LTBI) using tuberculin skin testing (TST) and asked about past TB disease. We assessed risk of LTBI and past history of TB disease associated with hospital employment. Among HCWs, we assessed risk associated with facility type (district hospital, referral hospital, health center), work setting (inpatient, outpatient), and occupation.

**Results:**

Age, gender, and HIV status was similar between the enrolled 1,131 HCWs and 381 SWs. LTBI was more prevalent among HCWs (62%) than SWs (39%). Adjusted odds of a positive TST result were 2.71 (95% CI 2.01–3.67) times greater among HCWs than SWs. Among HCWs, there was no detectable difference between prevalence of LTBI according to facility type, work setting, or occupation.

**Conclusion:**

HCWs are at greater risk of LTBI, regardless of facility type, work setting, or occupation. The current status of TB infection control practices should be evaluated in the entire workforce in all Rwandan healthcare facilities.

## Introduction

Tuberculosis (TB) is the most common opportunistic infection and leading cause of death in people living with HIV (PLHIV) [1–3]. In some resource-limited settings, up to 25% of PLHIV were found to have active TB disease upon initial presentation to an HIV care and treatment clinic [4]. Annual incidence of TB in Rwanda is 89 cases per 100,000 population and approximately 30% of persons diagnosed with TB also have HIV infection [5].

In high TB burden settings, health care workers (HCWs) are known to be at increased risk for latent TB infection (LTBI) due to their routine, sustained occupational exposure [6]. TB transmission in health care facilities in resource-limited settings is due to high TB prevalence, large numbers of patients seeking treatment, prolonged hospitalization, lack of isolation wards, overcrowding of admission wards, and weak or inadequate infection control (IC) measures [7–8].

Due to the association between HIV infection and TB and the emergence of multidrug-resistant (MDR) TB strains, TB IC activities have focused on settings where PLHIV receive services, such as HIV care and treatment clinics [9]. This targeted approach has been further justified by reports of nosocomial transmission during outbreaks of MDR TB [10–13]. However, recent reports have illustrated that TB risk is widespread in health care settings, supporting a more holistic approach to TB IC [14–19].

To date, there are no data available regarding the burden and risk of LTBI associated with Rwandan health care facilities. This study aimed to quantify the risk of LTBI, and past TB disease associated with work in outpatient and inpatient settings in health care facilities in Kigali Province in order to establish a baseline for monitoring and evaluation and to guide national TB control efforts in developing appropriate policies and procedures to effectively mitigate TB risk.

## Methods

We conducted a cross-sectional survey of LTBI and TB disease amongst HCWs and school workers (SWs) from October 2010 to December 2010, in Kigali City, Rwanda. We randomly selected seven of 17 health centers in Kigali, and purposefully included two of three Kigali District Hospitals (the military hospital of Kicukiro District was excluded), and one public reference hospital in Kigali (University Teaching Hospital of Kigali, CHUK). We purposively selected these facilities because there were few hospitals and all of them employ large numbers of health care workers. The military district hospital did not participate due to concerns from hospital leadership regarding participation in the study by members of the Rwandan military.

Staffing levels, patient loads and TB diagnostic methods of the facilities included in this study are described in S1 Table. Although specific IC practices within each facility were not evaluated as part of this study, commonly reported IC practices in Kigali at the time of this study included having a TB IC plan, triage and separation of patients with a cough and patients with known smear-positive TB, promoting cough hygiene, and keeping windows and doors open when possible.

The total site sample was 10 health facilities and 10 matched public schools from Kigali. The locations of the health facilities were used to define informal local catchment areas. From within each of those health care catchment areas, the nearest public school was included in the survey. Thus, workers from the selected schools within each catchment area serve as the reference from which we assessed the comparative risks of LTBI and TB disease among the health care workers within the respective catchment areas. We recruited school workers as our control group because they are socio-demographically similar to health care workers but differ with regards to workplace exposure to TB.

We recruited all clinical and nonclinical staff from health care facilities and teachers and administrators employed at participating schools. All HCWs and SWs must have served in the facility for at least 6 months and be 21 years of age to be eligible. All participants provided a written informed consent.

### Screening for TB and LTBI

Participants underwent a TB screening based on the Rwandan national TB screening questionnaire. The TB screening tool included the following elements: current cough (and duration); unintended weight loss > 3 kg during the last 4 weeks; night sweats ≥ 3 weeks; fever ≥ 3 weeks; close contact with a known TB patient during the last 2 years; and TB treatment during the last five years. Participants who reported a cough, unintended weight loss, night sweats, or fever were referred to a clinician for further evaluation. Prevalence of a past TB diagnosis was based on self-report. We used single-step Mantoux tuberculin skin tests (TST), with PPD RT23 to estimate prevalence of LTBI [20, 21]. TST placement was done by a team of nurses with previous experience in TST placement and reading. Prior to the study all study nurses were retrained in TST placement and reading by subject matter experts from the university teaching hospital of Kigali (UTHK and the Division of TB Elimination at CDC TST reactions were read between 48 and 72 hours after placement.

Induration of ≥10 mm was considered a positive result for HCWs and SWs; however a threshold of ≥5mm was used for PLHIV. Participants were given information on the management of a positive TB symptom screen and the need for further evaluation following a positive TST result.

### HIV status

Participants were asked if they knew their HIV status and, if so, whether testing had been performed within three months of the date of data collection. Self-reporting of HIV-positive status based on testing within the past three months was accepted as definitive. Those who did not know their HIV status and those that reported having a negative HIV test result from >3 three months ago were encouraged to undergo HIV screening using Determine test kits according to the national rapid testing algorithm. Participants were offered voluntary HIV counseling and testing services at the time of interviewing. The total number of PLHIV included those who self-reported positive status and those that tested HIV positive. For the purposes of this study, only those individuals who consented to HIV testing and returned negative test results were counted as HIV negative.

### Data collection and analysis

All participants were interviewed using a standardized questionnaire to gather information related to socio-demographic characteristics. As a proxy for socioeconomic status, we asked participants about the number of persons per room in their household. HCWs were asked additional questions regarding workplace related risk factors such as time employed as a HCW, amount of time spent working in outpatient and inpatient care, their specific occupation (i.e., doctor, janitor, nurse, administrator) and the specific departments within the health facility in which they worked.

The prevalence of LTBI was established and compared between those with assumed high exposure to TB represented by HCWs and those with reference exposure represented by SWs. Differences in prevalence of infection between HCW and SWs were attributed to the work place under the assumption that risk of TB infection outside the workplace was similar between HCWs and SWs. Descriptive characteristics of HCWs and SWs were quantified using cross tabulations, including differences and their 95% confidence intervals (CI). Conditional logistic regression was used to compare estimates of risk of LTBI and past TB disease between HCWs and SWs matched by health-care catchment area. Additionally, multiple logistic regression analysis was used to assess work-related risk factors for LTBI among HCWs; these risk factors included facility type (district hospital, referral hospital, health center), work setting (inpatient or outpatient), occupation (clinical, clinical support, auxiliary, and administrative), and department (high risk or standard risk). Clinical departments providing services to a patient population known to have a high prevalence of TB disease were considered high risk departments; these included HIV care and treatment and TB services. All other departments were considered standard risk departments. Risk factors for LTBI and TB disease were determined by calculating adjusted odd ratios (AOR) and 95% CIs after controlling for potential confounders such as age, gender, HIV status, and personal history of TB.

### Ethical Considerations

The protocol was approved by the former TRACPlus/MOH, the Rwanda National Ethics Committee, and the National AIDS Commission Review Board. In addition, the protocol was approved by the U.S. Centers for Disease Control and Prevention in Atlanta.

## Results

**Fig 1:**
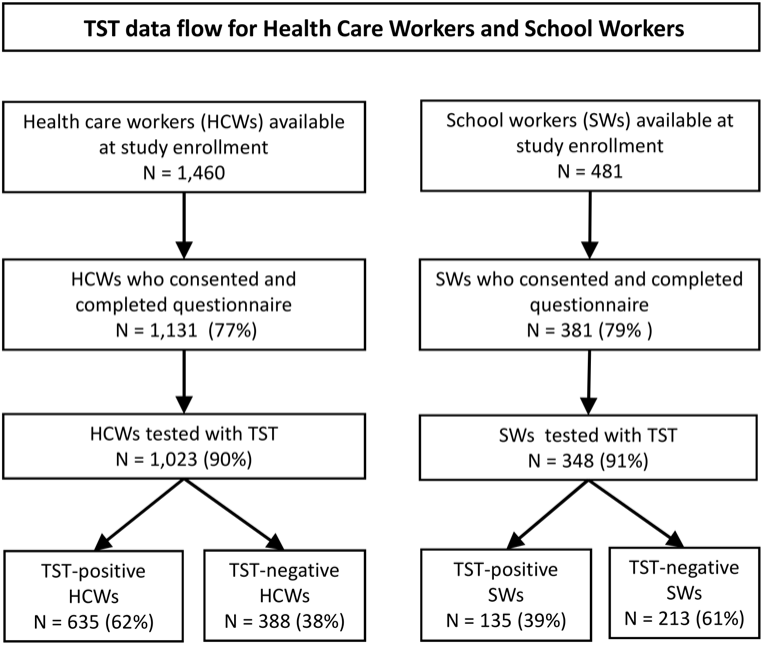
Data flow for health-care workers and school workers from ten health-care catchment areas in Kigali, Rwanda.

**Fig 2:**
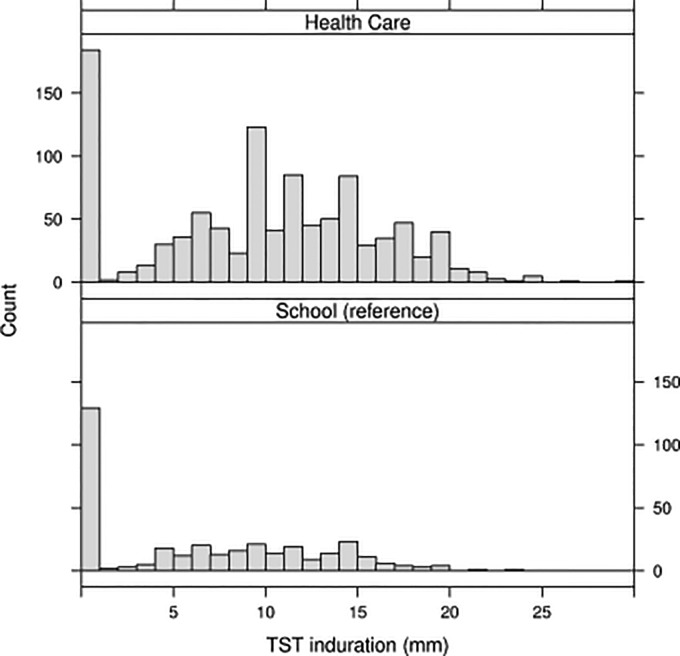
Distributions of TST indurations (mm) for health-care workers and school workers from ten health-care catchment areas in Kigali, Rwanda. Thirty (2.6%) and 10 (2.6%) HCWs and SWs, respectively, either self-reported or tested HIV positive, and 818 (99%) HCW and 280 (98%) SW who tested HIV-negative were classified as such.

Of 1,460 HCWs available at selected health facilities during the study enrollment period, 1,131 (77%) provided informed consent and completed the questionnaire; of these, 1,023 (91%) had a TST placed and read (Fig 1). Among 481 recruited SWs, 381 (79%) provided informed consent and completed the questionnaire; of these, 348 (91%) had a TST placed and read. The largest TST indurations occurred among the HCWs (Fig 2).

Apart from differences in work settings and personal history of TB disease, the populations of HCWs and SWs shared similar demographic characteristics. The median ages of HCWs and SWs were 32 and 34 years, respectively (Table 1). Approximately two-thirds of both groups were female. The proportions of HCWs and SWs who shared a residence with someone known to have been diagnosed with TB disease were nearly identical at 14.7 and 15.0%, respectively, and HIV prevalence was 2.6% in both groups. Socio-economic status, as measured by the number of persons per room in household, was similar between HCWs and SWs. The HCWs were more likely to have had TB disease (risk difference of 2.6%; 95% CI 0.9–4.3).

**Table 1:** Descriptive characteristics of 1,131 health care workers and 381 school workers from Kigali, Rwanda.

Characteristic	Health Care Workers	School Workers	Difference [95% CI]
Age, median years (range)	32 (21–78)	34 (21–71)	-3.5 [-4.8, -2.3]^1^
Female gender, n (%)	726 (64.2)	248 (65.1)	-0.9 [-6.4, 4.6)^2^
HIV status, n (%)			
Positive	30 (2.6)	10 (2.6)	0.0 [-2.3, 2.5]^2^
Negative	818 (72.3)	280 (73.5)	
Unknown	283 (25.0)	91 (23.9)	
Personal history of TB, n (%)			
Yes	47 (4.2)	6 (1.6)	2.6 [0.9, 4.3]
No	1,083 (95.8)	375 (98.4)	
Unknown	1 (0.1)	0	
Ever had household contact with TB, n (%)			
Yes	166 (14.7)	57 (15.0)	-0.7 [-4.9, 3.5]^2^
No	954 (84.3)	310 (81.4)	
Unknown	11 (1.0)	14 (3.7)	
Median number of persons/room in household (range)	1.3 (0.1–6.0)	1.4 (0.4–16.5)	0.1 [0.0, 0.2]^1^

^1^Difference between means.

^2^Risk difference (%) between positive/affirmative, excluding unknowns.

The prevalence of LTBI was higher in HCWs (62.1%) than SWs (38.8%), and the adjusted odds of a positive TST were 2.71 times greater among the HCWs (95% CI 2.01–3.67) after adjusting for age, gender, HIV status, contact with an active TB patient, and household density (Table 2). Secondarily, the odds of LTBI were increased by 4% (AOR 1.04; 95% CI 1.02–1.05%.) per year of age across both work settings, but there was no evidence of association between LTBI and gender, HIV status, personal contact with TB cases or the number of persons per room in households.

**Table 2:** Odds ratios for latent TB infection, as identified by TST results, among health-care workers relative to school workers from Kigali, Rwanda, before and after adjusting for potential confounders.

Characteristic	Valid TST results	Number (%) TST-positive	Unadjusted odds ratio [95% CI]	Adjusted odds ratio [95% CI]
Occupation				
Health-Care Workers	1,023	635	2.47	2.71
		(62.1)	[1.86, 3.27]	[2.01, 3.67]
School Workers	348	135	Reference	Reference
		(38.8)		
Age^1^			1.03	1.04
			[1.02, 1.04]	[1.02, 1.05]
Gender				
Male	494	272	0.90	0.98
		(55.1)	[0.72, 1.13]	[0.77, 1.24]
Female	877	498	Reference	Reference
		(56.8)		
HIV Status				
Positive	40	24	1.28	1.16
		(60.0)	[0.64, 2.56]	[0.57, 2.37]
Negative	1,098	569	Reference	Reference
		(51.8)		
Unknown	374	177	0.99	0.94
		(47.3)	[0.76, 1.28]	[0.72, 1.23]
Active TB Contact				
Yes	468	293	1.38	1.16
		(62.6)	[1.10, 1.75]	[0.90, 1.48]
No	850	449	Reference	Reference
		(52.8)		
Unknown	53	28	0.91	0.81
		(52.8)	[0.52, 1.60]	[0.45, 1.45]
Persons per Room in Household			0.93	0.91
			[0.80, 1.08]	[0.78, 1.08]

^1^Odds ratios for age/years represent the effects of each additional year of exposure. For example, an AOR of 1.04 implies a 4% increase in odds per year.

The HCWs were more likely to have reported a history of TB disease than the SWs, and the adjusted odds of prior TB disease were 3.14 times greater among the HCWs than the SWs (95% CI 1.25–7.90) after adjusting for age, gender, HIV status, contact with an active TB patient, and household density (Table 3). Secondarily, there was a strong association between history of TB disease and HIV infection (AOR 6.62; 95% CI 2.54–17.78) across both work settings, but no evidence of association with age, gender, contact with active TB cases or number of persons per room in households.

**Table 3:** Odds ratios for history of TB disease among health-care workers relative to school workers from Kigali, Rwanda, before and after adjusting for potential confounders.

Characteristic	Self-reports	Past disease (%)	Unadjusted odds ratio [95% CI]	Adjusted odds ratio [95% CI]
Occupation				
Health-Care Workers	1,130	47	3.14	3.14
		(4.2)	[1,28, 7.66]	[1.25, 7.90]
School Workers	381	6	Reference	Reference
		(1.6)		
Age^1^			1.00	1.01
			[0.98, 1.03]	[0.98, 1.04]
Gender				
Male	538	16	0.80	0.90
		(3.0)	[0.43, 1.46]	[0.48, 1.69]
Female	973	37	Reference	Reference
		(3.8)		
HIV Status				
Positive	40	8	6.16	6.72
		(20.0)	[2.47, 15.36]	[2.54, 17.78]
Negative	1,098	30	Reference	Reference
		(2.7)		
Unknown	373	15	1.56	1.43
		(4.0)	[[0.82, 2.96]	[0.74, 2.73]
Active TB Contact				
Yes	510	20	1.31	1.01
		(3.9)	[0.73, 2.38]	[0.54, 1.87]
No	945	29	Reference	Reference
		(3.1)		
Unknown	56	4	2.85	2.53
		(7.1)	[0.95, 8.55]	[0.82, 7.79]
Persons per Room in Household			0.93	0.71
			[0.80, 1.08]	[0.44, 1.16]

^1^Odds ratios for age/years represent the effects of each additional year of exposure. For example, an AOR of 1.04 implies a 4% increase in odds per year.

Among health-care workers, the odds of LTBI increased by 3% (AOR 1.03; 95% CI 1.01–1.06) for each year worked in health care after adjusting for other putative risk factors (Table 4). In contrast, there was no evidence of association between LTBI and facility type, work setting, department, or occupation among the HCWs. Among the HCWs, history of TB disease was associated only with HIV status (AOR 11.15; 95% CI 3.72–33.40) (Table 5).

**Table 4:** Associations between latent TB infection, as identified by TST results, and presumptive risk factors for health facility workers from Kigali, Rwanda.

Characteristic	Valid TST results	Number (%) TST-positive	Unadjusted odds ratio [95% CI]	Adjusted odds ratio [95% CI]
Age^1^			1.03	1.02
			[1.02, 1.05]	[0.99, 1.04]
Gender				
Male	374	223	0.85	1.03
		(59.6)	[0.65, 1.10]	[0.76, 1.38]
Female	649	412	Reference	Reference
		(63.5)		
HIV Status				
Positive	29	17	0.84	0.89
		(58.6)	[0.39, 1.78]	[0.40, 1.99]
Negative	753	473	Reference	Reference
		(62.8)		
Unknown	241	145	0.89	0.87
		(60.2)	[0.66, 1.20]	[0.63, 1.19]
Facility Type				
District Hospital	225	144	1.15	1.38
		(64.0)	[0.78, 1.70]	[0.91, 2.10]
Referral Hospital	597	369	1.05	1.13
		(61.8)	[0.75, 1.45]	[0.78, 1.64]
Health Center	201	122	Reference	Reference
		(60.7)		
Years Worked in Health Care^1^			1.05	1.03
			[1.03, 1.07]	[1.01, 1.06]
Department Assignment				
High Risk	287	183	1.11	1.09
		(63.8)	[0.83, 1.47]	[0.80, 1.48]
Standard Risk	736	452	Reference	Reference
		(61.4)		
Work Setting				
Inpatient	262	150	0.69	0.64
		(57.2)	[0.46, 1.03]	[0.41, 1.01]
Outpatient	162	107	Reference	Reference
		(66.0)		
Both	542	338	0.85	0.87
		(62.3)	[0.59, 1.23]	[0.59, 1.27]
Neither	53	38	1.30	1.26
		(71.7)	[0.66, 2.57]	[0.62, 2.58]
Unknown	4	2	0.51	0.57
		(50.0)	[0.07, 3.75]	[0.08, 4.30]
Job Assignment				
Clinical	418	270	1.01	1.05
		(64.6)	[0.66, 1.54]	[0.66, 1.67]
Clinical Support	146	92	0.95	0.92
		(63.0)	[0.58, 1.55]	[0.54, 1.57]
Auxiliary	286	163	0.74	0.83
		(57.0)	[0.48, 1.13]	[0.53, 1.31]
Administrative	126	81	Reference	Reference
		(64.3)		
Unknown	47	29	0.89	0.98
		(61.7)	[0.45, 1.79]	[0.48, 1.99]

^1^Odds ratios for age/years represent the effects of each additional year of exposure. For example, an AOR of 1.02 implies a 2% increase in odds per year.

**Table 5:** Associations between self-reported history of TB disease and presumptive risk factors for health facility workers from Kigali, Rwanda.

Characteristic	Self-reports	Past disease (%)	Unadjusted odds ratio [95% CI]	Adjusted odds ratio [95% CI]
Age^1^			1.02	1.01
			[0.99, 1.05]	[0.97, 1.06]
Gender				
Male	405	15	0.83	1.31
		(3.7)	[0.44, 1.56]	[0.66, 2.63]
Female	725	32	Reference	Reference
		(4.4)		
HIV Status				
Positive	30	7	9.27	11.15
		(23.3)	[3.65, 23.54]	[3.72, 33.40]
Negative	818	26	Reference	Reference
		(3.2)		
Unknown	282	14	1.59	1.31
		(5.0)	[0.82, 3.09]	[0.66, 2.63]
Facility Type				
District Hospital	289	17	1.02	1.70
		(5.9)	[0.49, 2.15]	[0.72, 4.05]
Referral Hospital	615	17	0.47	0.67
		(2.8)	[0.22, 0.97]	[0.27, 1.65]
Health Center	226	13	Reference	Reference
		(5.7)		
Years Worked in Health Care^1^			1.01	1.00
			[0.98, 1.05]	[0.95, 1.06]
Department Assignment				
High Risk	331	17	1.39	1.33
		(5.1)	[0.75, 2.55]	[0.68, 2.60]
Standard Risk	799	30	Reference	Reference
		(3.7)		
Work Setting				
Inpatient	282	12	0.69	0.86
		(4.3)	[0.30, 1.59]	[0.33, 2.21]
Outpatient	181	11	Reference	Reference
		(6.1)		
Both	605	21	0.56	0.56
		(3.5)	[0.26, 1.18]	[0.26, 1.23]
Neither	57	3	0.86	1.02
		(5.3)	[0.23, 3.19]	[0.29, 4.98]
Unknown	5	0	-	1.00
		(0)		-
Job Assignment				
Clinical	472	20	0.71	0.74
		(4.2)	[0.31, 1.66]	[0.28, 1.96]
Clinical Support	158	9	0.97	0.75
		(5.7)	[0.36, 2.60]	[0.25, 2.25]
Auxiliary	315	8	0.42	0.38
		(2.5)	[0.15, 1.14]	[0.13, 1.10]
Administrative	137	8	Reference	Reference
		(5.8)		
Unknown	48	2	0.70	0.83
		(4.2)	[0.14, 3,42]	[0.16, 4.34]

^1^Odds ratios for age/years represent the effects of each additional year of exposure. For example, an AOR of 1.02 implies a 2% increase in odds per year.

## Discussion

Since 2010 there has been no change in the national TB IC policy and implementation Guidelines, and therefore, we feel our findings are still representative of the current situation.

After controlling for risk factors for LTBI which are not job-related, and HIV infection, we found that rates of LTBI and past TB disease were both higher among health care workers than local controls.

Within health care settings, the risk of infection did not differ significantly across work locations and occupations. This finding is consistent with previously published literature and suggests increased transmission of TB within health care facilities in Kigali [15–16], and suggests that risk is incurred by duration of occupancy in health facilities, regardless of job occupation or department type. Other studies among HCWs from Africa that measured prevalence of LTBI found rates ranging from 33% in South Africa to 79% in Côte d'Ivoire [22–23]. Interestingly, investigators from nearby Uganda noted a nearly identical prevalence of LTBI among HCWs of 57% [24].

Previously published research has noted multiple, however, not always consistent, occupational risk factors for LTBI among HCWs in low- and middle-income countries [15]. Identified high-risk locations for LTBI within facilities include TB clinics, infectious disease wards, pulmonary wards, medical wards, and surgical/obstetric wards [25–29]. Previously identified high-risk occupations include radiology technicians, nurses, physicians, laboratory technicians, housekeepers, and maintenance personnel [27–31]. Unfortunately, because these studies used different definitions and different control groups, direct comparison to each other and to our study is difficult.

Our inability to identify high risk locations within health facilities is consistent with recent reports from some settings [30–31] and may be related to staff movement within the facility. If staff regularly move from working in one clinic location to another, then we would expect similar rates of infection across locations because the TST measures prevalence, not incidence of infection which may have occurred years ago when staff were working in a location different from their current work site. Although we were unable to assess the presence of administrative and environmental infection controls, the similar infection risk across work locations, settings, and occupations and within all facility types (Referral, District, and Health Center) suggests that key controls, such as ventilation, remain generally deficient.

The HIV prevalence among HCWs based on both HIV rapid testing and self-reports was slightly lower than the HIV prevalence among the general population in Rwanda (3.0% among persons 15–49 years old) [32]. In addition, HIV prevalence from both sources combined was considerably lower than the 2010 Demographic Health Survey HIV prevalence estimate for urban areas (7.1%) such as Kigali. The lower prevalence among HCWs and SWs may reflect the fact that a working population is healthier, thus less likely to have HIV, than the general population. Another explanation could be that HCWs and SWs were simply different from the rest of the Kigali population with regard to HIV risk (e.g., better educated regarding HIV acquisition and prevention measures and thus less likely to engage in high-risk behaviors).

Although not a study objective, we found it odd that we did not observe significant associations between TST reactions and sex or being a contact to an active case of TB. The fact that both men and women in our study shared similar workplace environments may have mitigated the association between TST reactions and sex. Because persons in regions with high rates of TB can never be certain that they were not exposed to someone with active TB, misclassification of exposure status might explain the lack of an association between being a contact to an active case and TST results. A potential limitation of our study was the use of TST to estimate prevalence of LTBI. The TST has been shown to cause false negative and false positive results, both of which could lead to imperfect measures of prevalence of LTBI [33].

In Kigali, Rwanda, nearly two out of every three HCWs was infected with TB. HCWs had almost three times the odds of LTBI as did controls from the same communities, even after controlling for socio-demographic factors. Our findings illustrate the elevated risk of TB involved with employment in a health care facility and indirectly suggest increased risk among patients visiting these same facilities, many of whom may be immune-compromised. Interestingly, the risk of LTBI was similar between staff working in outpatient and inpatient services, and including staff primarily assigned to HIV care and treatment and TB services.

The current status of TB infection control practices should be evaluated in the entire workforce in all Rwandan healthcare facilities. Establishing surveillance systems within health care facilities to monitor incidence of TB infection could help identify specific areas associated with highest risk and be used to identify persons who could benefit from isoniazid preventive therapy. Additionally, efforts should be made to monitor the outcomes of HCWs with TB, HIV, and TB/HIV [34]. Finally, although lower than that observed among HCWs, we noted that more than one in three SWs had LTBI, suggesting that strengthened infection control measures and active case finding are also needed outside of health care facilities.

## Supporting Information

S1 Table Characteristics of health facilities, Kigali Rwanda, including TB burden, and diagnostic methods. (DOCX)
