# Supplementary material for: Latent Tuberculosis Infection and Associated Factors among Health Care Workers in Kigali, Rwanda
Source: PLoS One. 2015 Apr 28;10(4):e0124485. doi: 10.1371/journal.pone.0124485 (PMC4412475; doi:10.1371/journal.pone.0124485)
Supplement: S1 Table — (DOCX) [file pone.0124485.s001.docx]

**S1 Table. Characteristics of health facilities, Kigali Rwanda, including TB burden, and diagnostic methods.**

|  | Approximate average size | | | |  |
| --- | --- | --- | --- | --- | --- |
| Facility Type | HCW | Daily Patient Load | Beds | TB patients/year | TB diagnostic methods |
| Health Centre (7: all are centers for HIV and TB diagnosis, treatment and care) | 42 | 40 | 30 | 40 | Sputum smear microscopy |
| District Hospital (2: both are centers for diagnosis and treatment of TB and HIV) | 200 | 60 | 150 | 70 | Sputum smear microscopy, chest X-ray, geneXpert, internal quality control of Health Centre sputum smears |
| University Teaching Hospital of Kigali | 700 | 80 | 300 | 100 | Sputum culture (solid and liquid media), chest-ray, geneXpert, TST, internal quality control of district’s sputum smears, TB mycobacterium differentiation (DNA sequencing) |
